# P-1537. Comparative effectiveness of early treatment with ceftolozane/tazobactam (C/T) relative to polymyxin (PB)-based therapy for non-COVID-19 patients (pts) with pneumonia (PNA) due to multi-drug resistant *Pseudomonas aeruginosa* (MDR-PSA) across US Hospitals

**DOI:** 10.1093/ofid/ofae631.1705

**Published:** 2025-01-29

**Authors:** Thomas Lodise, Jae Min, Brian H Nathanson, Yanbing Zhou, Emre Yucel

**Affiliations:** Albany College of Pharmacy and Health Sciences, Albany, New York; Merck, West Point, Pennsylvania; OptiStatim LLC, Longmeadow, Massachusetts; Merck, West Point, Pennsylvania; Merck & Co., Inc., North Wales, Pennsylvania

## Abstract

**Background:**

Ceftolozane/tazobactam (C/T) and PB are frequently used to treat adult pts with MDR-PSA PNA, but few clinical comparative studies exist. This study compared outcomes between non-COVID-19 hospitalized patients with MDR-PSA PNA who received early therapy with C/T- or PB-containing regimen.

**Figure 1.**
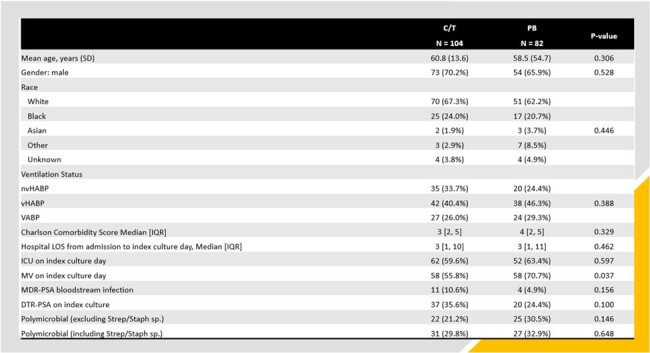
Comparison of Baseline Characteristics Between Patients Who Received Early Ceftolozane/Tazobactam or Polymyxin-Based Therapy Abbreviations: C/T: ceftolozane/tazobactam: PB; polymyxin: SD: standard deviation; LOS: nvHABP: non-ventilator hospital-acquired bacterial pneumonia; vHABP: ventilated hospital-acquired bacterial pneumonia; VABP: ventilator-associated bacterial pneumonia; length of stay; IQR: interquartile range; ICU: intensive care unit; MV: mechanical ventilation; MDR-PSA: multi-drug resistant P. aeruginosa; DTR: difficult-to-treat; Strep: Streptococcus sp.; Staph: Staphylococcus sp.

**Methods:**

Design: multi-centered observational study using 2016-22 data from the PINC AI Healthcare Database. Inclusion criteria: hospitalized; age ≥ 18 years; diagnosis for PNA; MDR-PSA on clinical culture site consistent with PNA diagnosis; receipt of C/T or PB within 3 days of index MDR-PSA culture; ≥2 consecutive days of C/T or PB; and no COVID-19 diagnosis. Outcomes: in-hospital mortality, hospital discharge destination (home vs other), recurrent MDR-PSA PNA, receipt of renal replacement therapy (RRT) post index MDR-PSA culture among pts who did not receive RRT prior to index culture day during their hospitalization (new RRT), and 30-day PNA-related readmissions. A rank order composite outcome was derived from the 5 individual study outcomes and a Desirability of Outcome Ranking (DOOR) analysis was performed to determine the probability of a more desirable outcome with C/T or PB (**Figure 1**).

**Figure 2.**
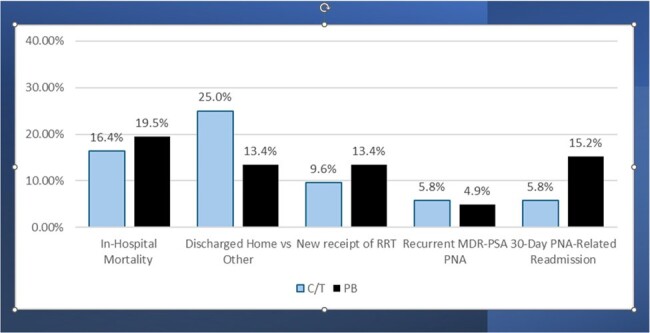
Comparison of Unadjusted Outcomes Between Patients Who Received Early Ceftolozane/Tazobactam or Polymyxin-Based Therapy Receipt of renal replacement therapy (RRT) post index MDR-PSA culture among pts who did not receive RRT prior to index culture day during their hospitalization (new RRT). Abbreviations: C/T: ceftolozane/tazobactam, PB: polymyxin; RRT: renal replacement therapy, MDR-PSA: multi-drug resistant P. aeruginosa, PNA: pneumonia.

**Results:**

During the study period, 186 pts met the study criteria (104 C/T pts and 82 PB pts). Most (90.2%) of PB pts received colistin. Treatment groups were largely comparable at baseline (**Figure 2**). In-hospital mortality rates were similar between groups but pts who received C/T versus PB were significantly more likely to be discharged to home (**Figure 3**). In the DOOR analysis, a C/T-treated pt had a significantly higher probability of a more favorable outcome than a PB-treated pt (DOOR Probability: 57.7% (95% CI: 50.1%, 65.3%). In the DOOR partial credit analysis (**Figure 4**), the mean partial credit score was significantly higher in in the C/T vs PB group for scenario B (pts who place more value on minimizing events and do not accept any undesirable event).

**Figure 3.**
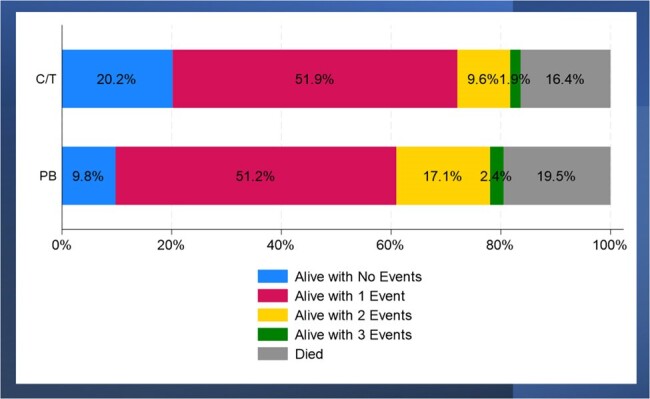
Comparison of Desirability of Outcome Ranking (DOOR) Outcome Between Patients Who Received Early Ceftolozane/Tazobactam or Polymyxin-Based Therapy DOOR Probability was 57.7% (95% CI: 50.1%, 65.3%), indicating there was a more desirable outcome in C/T-treated patients relative to CZA-treated patients. Rank 1 represented the most desirable outcome and included anyone who was discharged alive to home and did not experience any of the undesirable, pre-specified events. Rank 6 represented the least desirable outcome and included all patients who died during their hospitalization. Ranks 2 through 5 include patients who were discharged alive but had 1, 2, 3, or 4 events, respectively. The events included in the DOOR analysis were as follows: not discharged home, receipt of renal replacement therapy (RRT) post index MDR-PSA culture among pts who did not receive RRT prior to index culture day during their hospitalization (new RRT), recurrent MDR-PSA PNA, and 30-day PNA/sepsis-related readmission.

**Conclusion:**

The findings suggest pts with MDR-PSA PNA who receive early treatment with C/T have a higher probability of a more favorable outcome than pts who receive PB. Further large-scale studies are necessary to comprehensively understand the outcomes associated with these treatments for MDR-PSA PNA.

**Figure 4.**
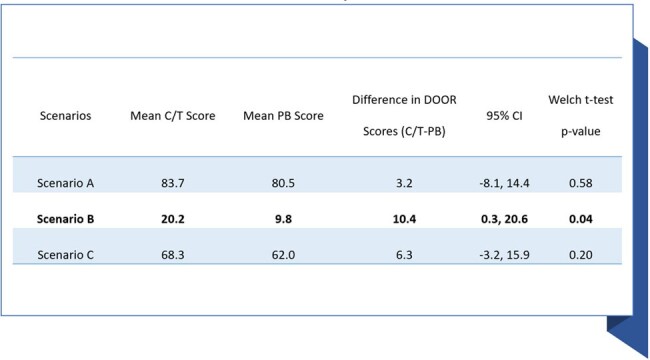
Comparison of Desirability of Outcome Ranking (DOOR) Partial Credit Scores Between Patients Who Received Early Ceftolozane/Tazobactam or Polymyxin-Based Therapy Scenario A represents a patient who values only hospital survival (equivalent to a mortality outcome). Scenario B represents a patient who places more value on minimizing events and would not accept any undesirable event. Scenario C represents a patient who places significant value on survival but balances this with wanting to avoid some events. For each scenario, the mean of the partial credits scores is calculated for each treatment group and then the difference between groups is obtained. A difference with a 95% CI that overlaps zero indicates no significant difference between groups. Abbreviations: CI, confidence interval; DOOR, desirability of outcome ranking C/T, ceftolozane/tazobactam; PB, polymyxin.

**Disclosures:**

**Thomas Lodise, Jr., Pharm.D., PhD**, MERCK: Advisor/Consultant **Jae Min, PhD**, MERCK: Stocks/Bonds (Public Company) **Brian H. Nathanson, Ph.D.**, MERCK: Advisor/Consultant **Yanbing Zhou, PhD**, Merck: I am a full time Merck Employee and own stocks in the retirement plan provided by Merck.|Merck: Stocks/Bonds (Public Company) **Emre Yucel, PhD**, Merck: I am a full time Merck Employee and own stocks in the retirement plan provided by Merck.|Merck: Stocks/Bonds (Public Company)

